# Waste valorization utilizing green nanotechnology: a sustainable approach for pomegranate peel agro wastes in skincare formulations

**DOI:** 10.1186/s40643-025-00958-6

**Published:** 2025-12-24

**Authors:** Mousumi Debnath, Dikshita Aneja

**Affiliations:** Department of Biosciences, Manipal University Jaipur, Jaipur, Rajasthan 303007 India

**Keywords:** Sustainability, Waste valorization, Pomegranate peels, Circular economy, Green nanotechnology, Bioactive components, Skincare formulations

## Abstract

Pomegranate peels, a valuable waste byproduct, aligned with the Sustainable Development Goals by United Nations offering a possibility for use as a raw material in herbal formulations. The review aims to highlight the pharmaceutical importance of pomegranate peel bioactive compounds and their applications in the skincare industry. A systematic search of studies identified through scientific databases resulted in the PRISMA chart. Additionally, an exhaustive search using keywords such as pomegranate peel, skincare, ADME, nanoparticles, therapeutics, and bioactive compounds led to a bibliometric analysis. The peels support the waste-to-wealth concept with a rich repertoire of flavonoids, including quercetin, kaempferol, and catechin, as well as polyphenolic compounds and other tannin compounds, such as punicalagin and ellagic acid. These compounds serve as natural reducing agents for synthesizing various nanoparticles and unveiling therapeutic activities, including antibacterial, anti-inflammatory, anticancer, and antioxidant properties. Furthermore, an attempt to bridge the gap by examining the pharmacokinetic studies on key bioactive compounds identified through ADME analysis, supported the transdermal and topical applications of these compounds. The profiling suggests that tannin compounds, such as punicalagin, have low skin permeability, indicating potential roles in topical applications. In contrast, ellagic acid exhibits high skin permeability, suggesting possible applications in products such as sunscreens. The combinatorial formulation, consisting of pomegranate peel-derived nanoparticles, has potential additional benefits by facilitating skincare products with photoprotection, enhanced hydration, and topical applications. The study also calls for future research focused on clinical validation and scalable commercial applications.

**Graphical Abstract:**

**Figure:**
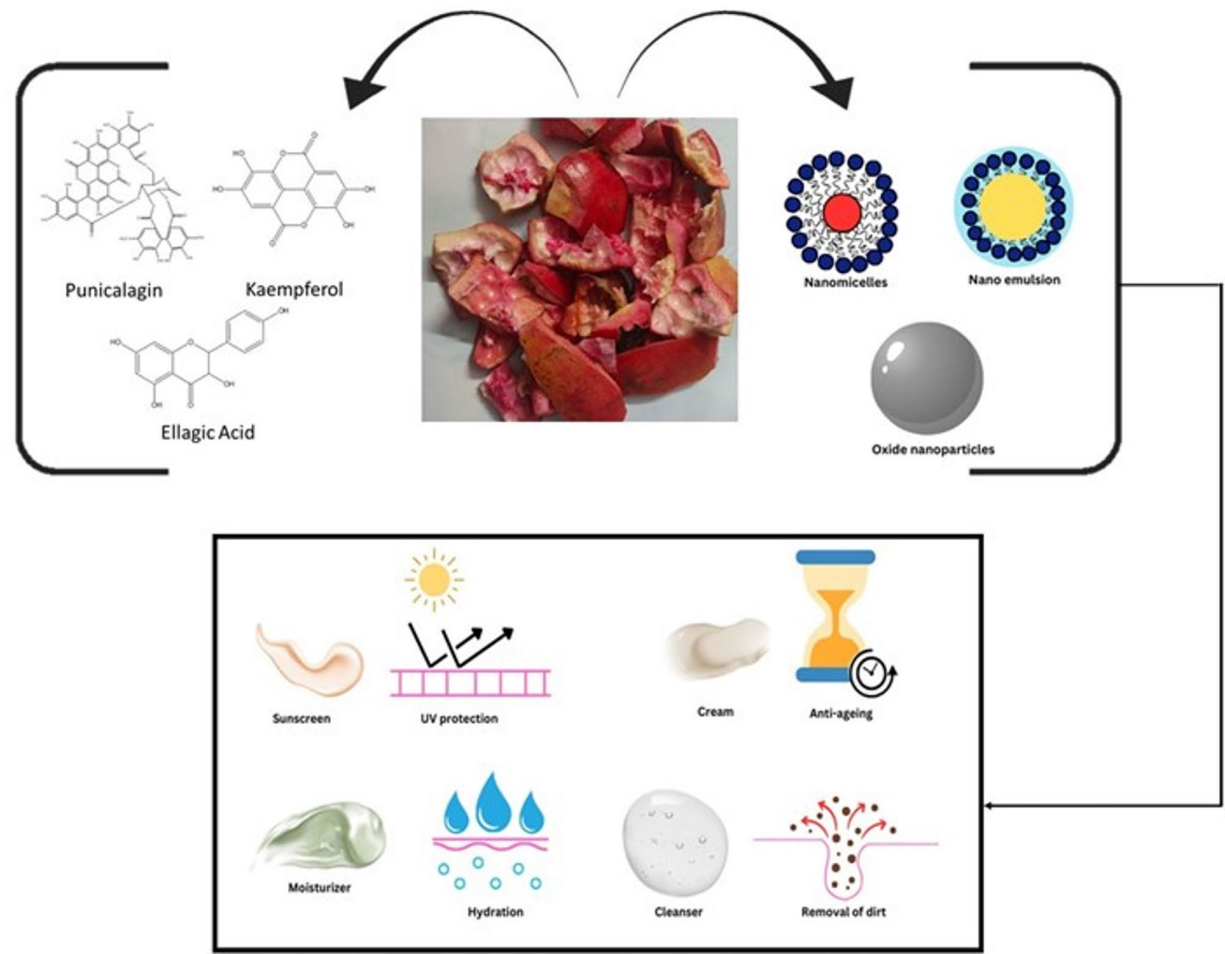

**Supplementary Information:**

The online version contains supplementary material available at 10.1186/s40643-025-00958-6.

## Introduction

Reduce, recycle, and reuse are the 3Rs principle in the policy of the Food waste management hierarchy employed in various countries across the globe (Saparbekova et al. 2023). These policies also focus on reusing intangible plant components, such as organic waste, which is usually discarded during industrial processing. One way to reuse this type of waste is by creating value-added formulations that can benefit industrial applications. Also, the overproduction of waste can be related to the overgrowing urban population (Su et al. 2024). The annual production of agricultural wastes is increasing rapidly at a 5 to 10% rate, leading to various environmental concerns (Afzaal et al. 2022; Riseh et al. 2024). One such waste that is generated in massive amounts is pomegranate peels. Inedible pomegranate skin makes up as much as 40% of the fruit’s weight (Vučić et al. 2019). Food processing plants frequently face challenges in disposing of their waste, with many of these, including pomegranate peels, being regarded as invaluable nutraceuticals for potential use in diverse food formulations (Goula and Lazarides 2015). The United Nations established the 17 Sustainable Development Goals (SDGs), which encompass goals and targets designed to achieve sustainable development. There is an emphasis on environmental sustainability and waste management. Combining the 3Rs policy and resource efficiency, the aspects of a sustainable circular economy aim to support the agenda of conserving natural resources (Khajuria 2021).

Pomegranate, or *Punica granatum* L., belongs to the genus *Punica* and the family Lythraceae (previously named Punicaceae). The Latin terms *Pomum*, which means apple, and *granatus*, which means grainy, are combined to form the name *Punica granatum* L., which means seeded apple (Jawad et al. 2013). Originating from Iran, this fruit is widely cultivated across various Asian and European regions (Villa-Ruano et al. 2020). This fruit contains bioactive components, including anthocyanins, polyphenols, and tannins, making it a functional food with various health benefits (Pirzadeh et al. 2021). Recent studies have demonstrated the presence of high concentrations of multiple phytochemicals and bioactive substances, including polyphenols, hydrolyzable tannins, and flavonoids, in pomegranate peels (Chen et al. 2020). As a waste byproduct, peels are rich in powerful antioxidants that help combat various diseases. Moreover, they are also shown to reduce oxidative stress, lower cholesterol, blood pressure, and improve heart health (Ain et al. 2023). Pomegranate peel has also been found to be an effective therapeutic against gastrointestinal diseases (Parisio et al. 2020). Pomegranate peels contain a wide range of secondary metabolites, including punicalagin and ellagic acid, which significantly contribute to their therapeutic value. All these values of the peels contribute to SDG 3 (Good Health and Well-being). The various properties of pomegranate peel render it a valuable byproduct for diverse applications across several industries. These include its use as nutraceuticals and in food processing within the food sector, as well as its role in the cosmetics, skincare industry and pharmaceuticals. Additionally, it aligns with the “waste to wealth” approach, effectively addressing environmental concerns.

A productive approach for managing biodegradable waste is to utilize it as a raw material for the green synthesis of nanoparticles (Aswathi et al. 2023). Nanotechnology represents a scientific domain focused on creating materials and structures at the nanoscale, ranging in size from 1 to 100 nanometres, using diverse synthesis strategies (Jamkhande et al. 2019). Currently, there is an unexpected rise in the use of nanoparticles across multiple disciplines, such as organic and inorganic chemistry, molecular biology, physics, medicine, and materials science (Jamkhande et al. 2019). The primary reason for this growing interest is the high surface-to-volume ratio of nanoparticles, which also results in high surface energy. As the surface energy increases, reactivity also increases, thereby making nanoparticles highly versatile for various applications (Jadoun et al. 2021). Multiple approaches, including chemical, physical, and green synthesis, are utilized to prepare various nanoparticles (Gour and Jain 2019). In comparison to the synthetic approach, the green synthesis approach has significant advantages (Duan et al. 2015). The green method for nanoparticle synthesis is non-toxic, rapid, effective, clean, and environmentally friendly technology commonly employed to synthesize metal oxide and metallic nanoparticles (Dhand et al. 2015). The innovative aspect of using these green-synthesized pomegranate peel-mediated nanoparticles is their incorporation into specific formulations that can help enhance and improve skin health. Pomegranate peels have the potential to be utilized in cosmeceutical formulations for skin health due to their inherent antioxidant properties. In human reconstituted skin, pomegranate formulations have been shown to increase levels of proliferating cell nuclear antigen and tropoelastin, while also blocking UV-B-driven DNA damage (Kandylis and Kokkinomagoulos 2020). The punicic acid in pomegranate peel has been shown to offer protective properties for the skin’s collagen fibres, which hasten wound healing and lessen the visibility of scars. According to Ain et al. (2023), punicic acid also has anti-inflammatory qualities. It reduces inflammation and oxidative damage by promoting the production of the peroxisome proliferator-activated receptor (Ain et al. 2023).

Additionally, it has several applications in the food sector, including the development of valuable formulations, food packaging, and preservation (Kandylis and Kokkinomagoulos 2020; Singh et al. 2023b). Due to its explicit antioxidant and antimicrobial properties, it can be utilised as a preservative, improving both the shelf life and quality of food formulations, and as a nutritional enhancer (Singh et al. 2023b). Pomegranate peels, often discarded as waste, contain valuable uses. They can be turned into biochar for cleaning dyes from water. They are also used as natural food additives in the food industry and offer benefits in cosmetics (de Souza Mesquita et al. 2024; Maggiore and Setti 2025; Mahmoud and Azab 2025). These studies show how we can reduce waste by repurposing pomegranate peels into valuable products. This process not only helps reduce waste but also supports the Sustainable Development Goals (SDGs) by promoting circular economy practices and enhancing resource efficiency. These initiatives contribute to achieve SDG 9, SDG 3, and SDG 13.

Furthermore, these approaches not only foster sustainability but also cater to the increasing demand for eco-friendly formulations among consumers (Sarkar et al. 2024). By embracing innovative extraction methods and utilizing products enriched with peels, companies can significantly expand their market share (Noreen et al. 2025). The large-scale extraction of bioactive compounds from peel waste presents a substantial opportunity to reduce manufacturing costs, ultimately improving the overall economics for various industries (Pereira et al. 2025). The global market for pomegranates is expanding due to a growing trend in health-conscious consumption. In fact, pomegranate peels have numerous benefits that contribute to their valuable market presence across various food industries. These market trends not only emphasize the use of natural substances but also enhance consumer perceptions by providing innovative and health-beneficial products (Siddiqui et al. 2024).

The large-scale extraction of bioactive compounds from peel waste presents a substantial opportunity to reduce manufacturing costs, ultimately improving the overall economics for various industries. It can be used to formulate various value-added products. Additionally, these products can be more cost-effective than traditional ones. In the food industry, peels present considerable potential. These peels are utilized as a natural food additive, enhancing the quality, flavour, color, and texture of food products. Recent research has demonstrated their effectiveness as a natural preservative, showing that peel extracts can extend the shelf life of perishable items, such as fish, by combating food spoilage. The study reveals that the primary factors contributing to this effectiveness are their notable antimicrobial and antioxidant properties (Hossain et al. 2023). Furthermore, pomegranate peels can be used to create sustainable antimicrobial food packaging. The life cycle assessment of these packaging films indicates a 59% reduction in environmental impact, highlighting their eco-friendly benefits (Baniasadi et al. 2025). In the cosmetic industry, they can be used in the preparation of formulations that tend to show UV protection benefits (Tumbarski et al. 2025). By scaling up the extraction processes and developing these products, it creates employment and job opportunities in industries. Additionally, applying green extraction methods such as microwave-assisted, ultrasound-assisted, and hydrodynamic cavitation-assisted methods. These methods can help reduce environmental impact and energy consumption (Ballistreri et al. 2024). These scaling-up processes require regulatory compliance for the products to be launched into the market.

This review offers an in-depth understanding of the functional and therapeutic potential of pomegranate peels, a valuable yet underutilized resource. It highlights the bioactive compounds present in the peel and their potential industrial applications, including the development of value-added formulations. The study also reviews the potential of developing value-added skin care formulations utilizing non-toxic nanoparticles derived from pomegranate peels. Furthermore, by leveraging the antioxidant and anti-aging properties of pomegranate peel extracts, this approach not only enhances skin health but also supports waste reduction, sustainable development, and innovation in the skincare industry .

## Methodology

**Fig. 1:**
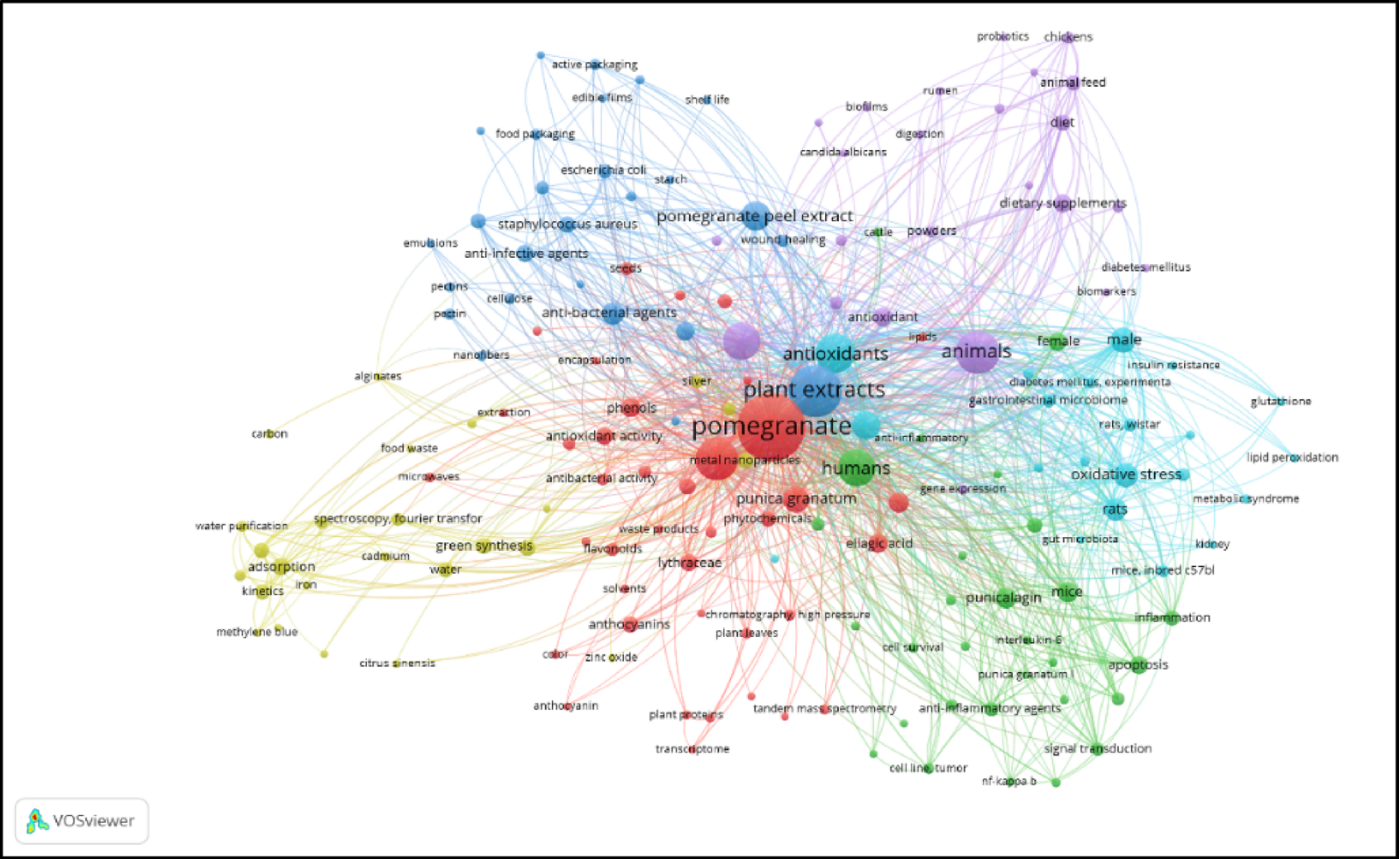
Bibliometric analysis of pomegranate peel

The systematic review was conducted using various scientific search engines, including PubMed, Google Scholar, ScienceDirect, Web of Science, and Scopus, with keywords such as “pomegranate peel”, “pomegranate peel therapeutics”, “pomegranate bioactive compounds”, “pomegranate peel and skincare”, “pomegranate peel nanoparticles”, and “pomegranate peel ADME studies" . Based on publication data and an analysis of trends, patterns, and gaps in studies, a discrete pictorial description was created, as depicted in Fig.1 . This bibliometric analysis illustrates the relationship between scientific publications on pomegranate peel, focusing on the interrelationships within research areas, as shown with different colors. It clearly showed that pomogrante peel extracts are in high demand, and their use in therapeutics has been well researched. The molecular mechanism and their signal transduction pathways of specific bioactive compounds have also been studied. There are indications that animal studies on both male and female animals have been conducted specifically on Wistar rats and mice. Research has been conducted in the fields of antibacterial, diabetes, gut microbiota, inflammation, and cancer. There are also some reports on green synthesis and zinc oxide. Considering this data, a further search was conducted to formulate the PRISMA chart .

**Fig. 2:**
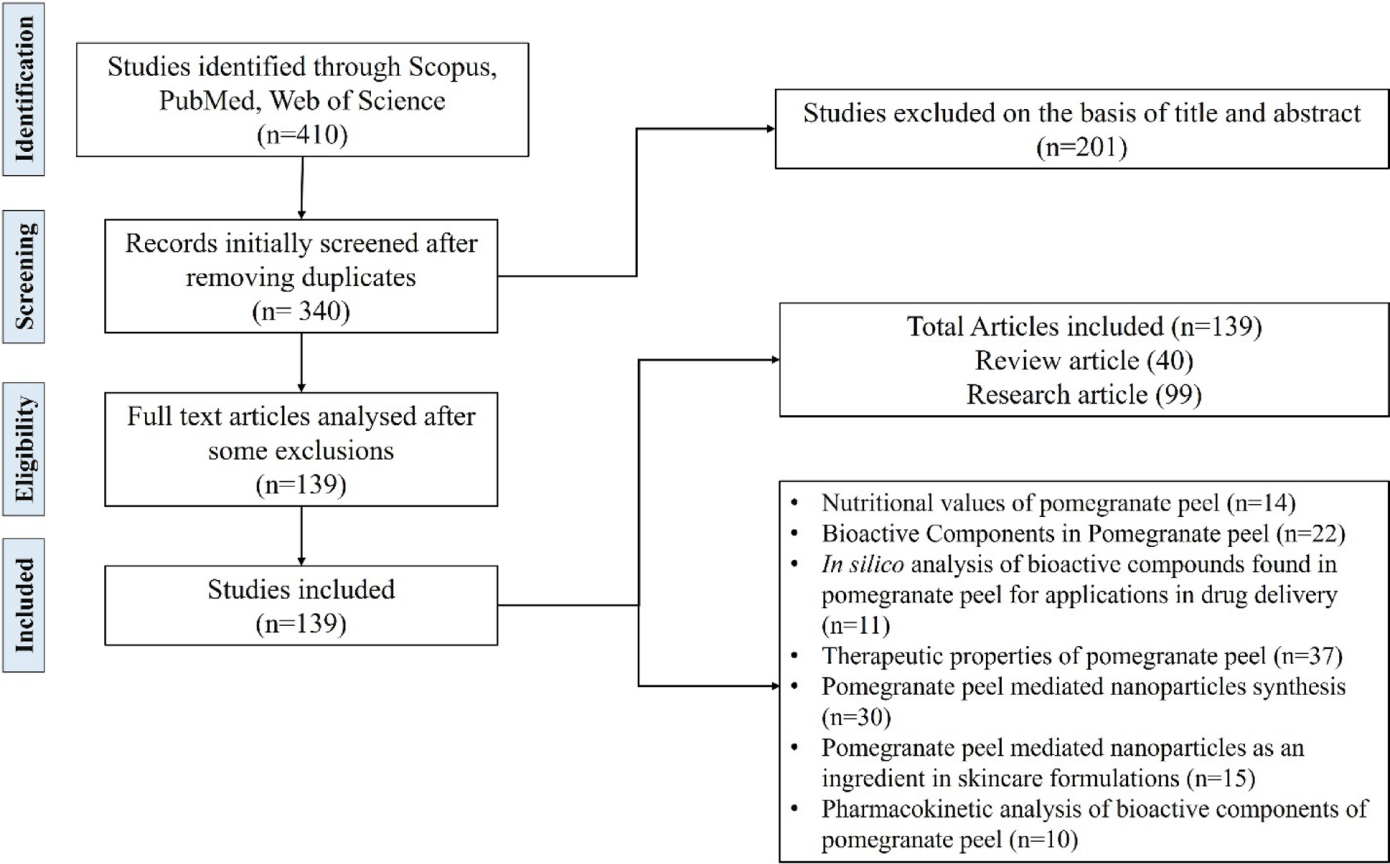
PRISMA chart representing the systematic pattern of the methodology of the study

After an initial search, some records were screened, and a total of 340 works were selected. Furthermore, based on the abstract and title, 201 records were excluded. Consequently, by bridging the gaps and highlighting the identified searches, the remaining works were selected to prepare the module for this proposed comprehensive review work, as shown in the flow chart in Fig. 2.

## Nutritional values of pomegranate peel

**Fig. 3:**
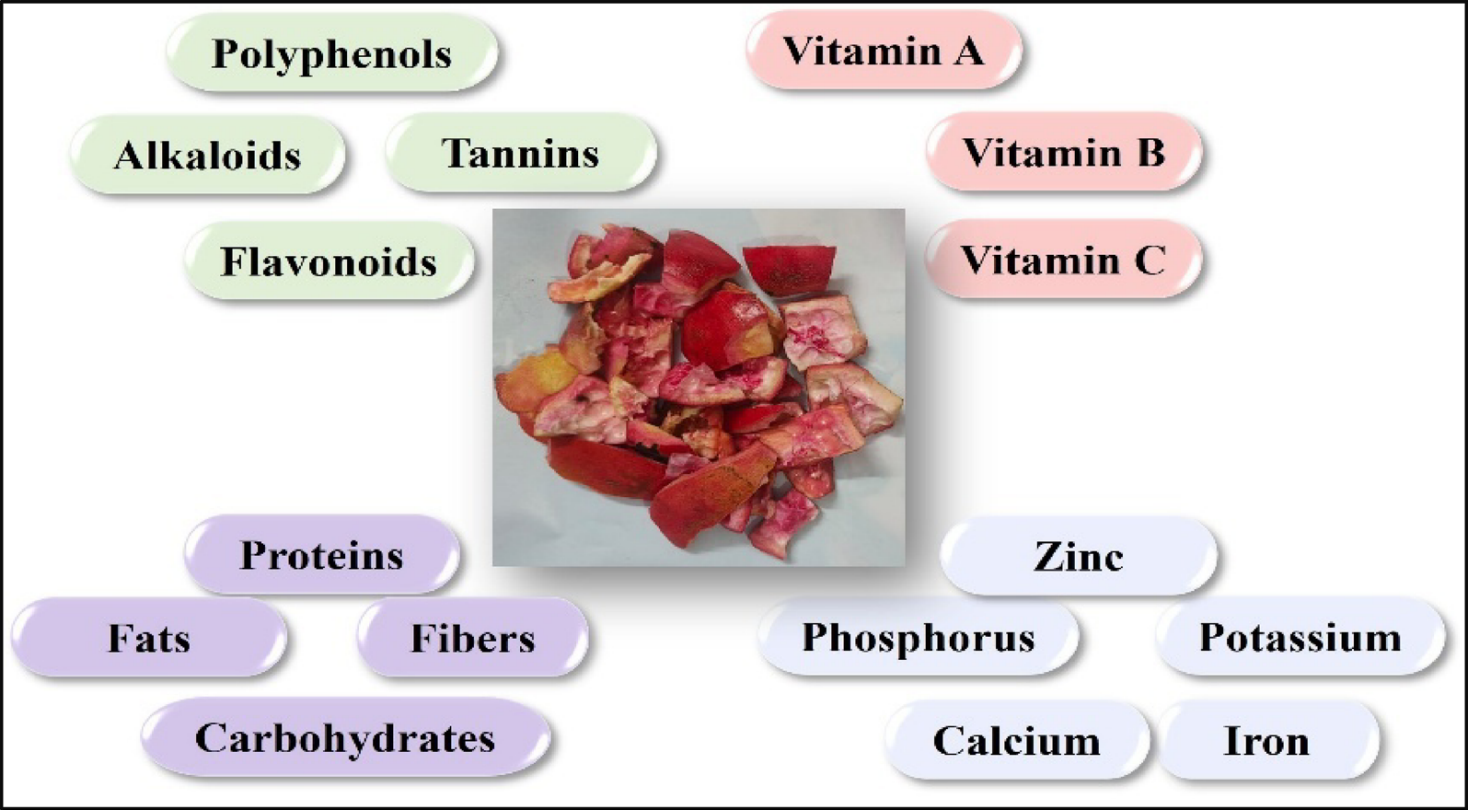
Nutritional and phytochemical composition of pomegranate peels illustrating the presence of polyphenols, flavonoids, vitamins, and essential minerals

Pomegranate is a highly nutritious fruit known for its health benefits. Besides its consumption as a fresh fruit, it is often processed into a variety of beverages. During processing, a significant amount of waste is generated, with peels accounting for more than 30% of the total waste. Peels, being a waste byproduct, contain a variety of bioactive components, proteins, fats, carbohydrates, fibres, and other nutritional compounds Fig. 3. From ancient times, peels have been used in various home remedies to treat colds and coughs. Research has shown the use of pomegranate peel in different food products and as a natural food additive (Kaderides et al. 2021). Research by Muhammad et al. (2023) examined the preparation of cookies fortified with pomegranate peels. They added the peel powders to wheat flour and made cookies that showed enhanced nutritional values as compared to the control ones (Muhammad et al. 2023).

They are a rich source of carbohydrates and crude fiber, varying from 50 to 70% and 5–20% per 100 g, respectively. The protein content is relatively low, ranging from 2 to 9%. It also contains a negligible amount of fat, ranging from 0 to 1%. Similarly, in a study, peels were incorporated into snack bars, which showed an increase in mineral, fat, total phenols, and total flavonoid content (Abbas et al. 2025). Pomegranate is grown widely all over the globe. Several reports on the nutritional content of pomegranate peel from different countries elucidate the variability, which can also be related to the efficacy of their therapeutic properties. Table 1 presents a brief comparison of the nutritional values found in pomegranate peels from different countries.

**Table 1:** A comparative analysis of the nutritional contents of pomegranate peel from different countries

S. No.	Site of study	Ash (%)	Protein (%)	Crude fiber (%)	Total fat (%)	Carbohydrate (%)	Total polyphenol (mg/g GAE)	Total flavonoids (mg/g QE)	DPPH scavenging activity (µg/mL)	References
1	Egypt	4.22	8.97	19.41	0.85	59.60	143.4	58.55	IC_50_-23.5	Morzelle et al. (2016)
2	India	4.32	3.74	17.31	0.85	66.51	269.9	10	91.52%	Malviya et al. (2014); Sun et al. (2021); Alam et al. (2022)
3	Bangladesh	2.41	8.23	7.75	4.04	63.11	178.61	64.64	66.86%	Imran et al. (2019); Čolić et al. (2022)
4	Sri Lanka	3.74	4.73	15.03	–	69.6	402	52.64	IC_50_-20.6	Baranwal et al. (2022)

QE- Quercetin equivalent, GAE- Gallic acid equivalents, IC_50_- The concentration at which 50% DPPH scavenging activity is observed

In addition to their unique composition, pomegranate peels are particularly rich in bioactive components, notably certain hydrolysable tannins such as ellagic acid, punicalagin, and other related derivatives. Numerous studies have indicated that the concentration of these bioactive compounds varies depending on the cultivar and processing methods used. For instance, a study analyzed two pomegranate peel cultivated varieties, *Ganesh* and *Bhagwa*, for the quantitative estimation of these components through HPLC. It also examined the effects of different drying methods (freeze, sun, and oven). The freeze-dried *Bhagwa* variety proved to be the most effective method, yielding punicalagin at 15.2 mg/g, ellagic acid at 13.6 mg/g, gallic acid at 32.2 mg/g, and quercetin at 2.5 mg/g (Kumar et al. 2022). Furthermore, a recent study investigated extraction methods, including ultrasound, microwave, and hydrodynamic cavitation, for processing fruits across several pomegranate cultivar varieties, including *Acco*,* Parfianka*,* Wonderful*,* Jolly Red*,* Valenciana*, and *Hicaz.* The findings revealed that hydrodynamic cavitation-based extraction yielded the highest amounts of ellagitannins for only one cultivar, *Wonderful*, with a 20% increase compared to other varieties and extraction methods (Ballistreri et al. 2024). For bioavailability, the key ellagitannin compounds, including ellagic acid and punicalagin, have limited or poor bioavailability due to their binding with certain proteins and minerals, which reduces their ability to undergo further chemical reactions in the body. Additionally, these ellagitannin compounds are generally broken down by weak acids, bases, and certain enzymes (Zam et al. 2025). These ellagitannins are hydrolysed into ellagic acid and its derivatives for absorption. For ellagic acid, it can be either absorbed directly into the small intestine or stomach, or if unabsorbed, it can be metabolized and converted into urolithins by gut microbiota. These urolithins are then circulated through the body’s circulatory system (Kang et al. 2016). To improve the bioavailability of ellagitannin compounds, nanoencapsulation can be utilized to reduce the size of bioactive molecules, enhancing their absorption and overall availability in the body (Venusova et al. 2021).

## Bioactive components in pomegranate peel

Pomegranate peel has been utilized since ancient times for treating various ailments and as a traditional remedy due to its content of beneficial compounds. It is known to contain polyphenols, flavonoids, and hydrolysable tannins. The bioactive compounds in pomegranate peels are known to have therapeutic effects, including anti-inflammatory, antioxidant, and anti-genotoxic properties. The health benefits mainly stem from the intense activity of water-soluble hydrolysable ellagitannins, especially punicalagin, which appears in two forms: α and β (Huang et al. 2025). The punicalagins are the primary components of peels that contribute to their antimicrobial, anti-inflammatory, anticancer, and antioxidant properties (Siddiqui et al. 2024). Additionally, the peels are a rich source of flavonoids, such as catechin, kaempferol, and quercetin, with catechin being particularly notable for its potential anticancer effects. The bioactive components in pomegranate peel exist in a complex mixture, and their synergistic effects contribute to various therapeutic properties, emphasizing the potential for enhanced health benefits compared to individual compounds (Mo et al. 2022). Recent research observed the synergistic effects of three tannin compounds found in peels: punicalagin, punicallin, and ellagic acid. This combination resulted in the downregulation of cytokines IL-6, IL-8, and TNF-α, demonstrating strong anti-inflammatory potential (Čolić et al. 2022). In another study, it was observed that the juice of pomogrante fruit comprises a blend of bioactive components, showing high antioxidant and antiproliferative activity as compared to the single use of commercial standards of purified punicalagin and ellagic acid, respectively (Seeram et al. 2005).

It prevents cell growth, induces cell cycle arrest, facilitates apoptosis, and regulates cell signalling pathways through various mechanisms (Du et al. 2025). A comprehensive list of bioactive components identified through an extensive review of the literature, along with their therapeutic benefits, is presented in Table 2.

**Table 2:** Bioactive components identified from pomegranate peel

S. No	Compound name	Structure	Bioactivity	References
Polyphenolic compounds				
1.	Pomegranatin A		Inhibited proliferation of *Candida albicans*	Ruan et al. (2022)
2.	Pomegranatin B		Inhibited proliferation of *Candida albicans*	Ruan et al. (2022)
3.	Pomegranatin C		Antioxidant and antifungal	Ruan et al. (2022)
4.	2,3-O-(S)-hexahydroxydiphenoyl (HHDP)-βD-glucopyranose		Antioxidant, antifungal, and antitumor efficacy against HeLa cell lines	Ruan et al. (2022)
5.	Gemin D		Antioxidant and antifungal	Ruan et al. (2022)
6.	Casuariin		Antifungal and antitumor efficacy against HeLa cell lines	Ruan et al. (2022)
7.	Gallic acid		Gastrointestinal, neuropsychological, cardioprotective	Morzelle et al. (2016)
8.	Caffeic acid		Antioxidant, anti-inflammatory, neuroprotective, anticancer	Alam et al. (2022)
9.	Punicacortein D		Antioxidant and antimicrobial	Ruan et al. (2022)
10.	Punicacortein C		Antioxidant, antifungal and antitumor efficacy against HeLa cell lines	Ruan et al. (2022)
11.	Granatin A		Antifungal and antitumor efficacy against HeLa cell lines	Ruan et al. (2022)
12.	Punicagranin A		Anti-inflammatory activity	Li et al. (2022)
13.	Punicagranin B		Anti-inflammatory activity	Li et al. (2022)
14.	Punicagranin C		Anti-inflammatory activity	Li et al. (2022)
15.	Punicagranin D		Anti-inflammatory activity	Li et al. (2022)
16.	Punicagranin E		Anti-inflammatory activity	Li et al. (2022)
17.	Punicagranin F		Anti-inflammatory activity	Li et al. (2022)
18.	Punicagranin G		Anti-inflammatory activity	Li et al. (2022)
19.	Punicagranin H		Anti-inflammatory activity	Li et al. (2022)
20.	Punicagranin I		Anti-inflammatory activity	Li et al. (2022)
21.	Isohydroxymatairesinol		Antimicrobial and anticancer	Nazeam et al. (2020)
Terpenoid glycoside				
22.	β-Sitosterol-3-O-glycoside		Antioxidant and analgesic	Sun et al. (2021)
Sterol				
23.	β-sitosterol		Anthelmintic, antimutagenic and antimicrobial	Sun et al. (2021)
Triterpenoid compounds				
24.	Ursolic acid		Hypolipidemic, anti-inflammatory, anti-cancer andantimicrobial	Sun et al. (2021)
25.	Corosolic acid (2α-hydroxyursolic acid)		Induce programmed death of cell in tumor cells	Sun et al. (2021)
26.	Asiatic acid		Induce apoptosis in tumor cells and antimicrobial	Sun et al. (2021)
27.	Brevifolin carboxylic acid		Treatment of hepatitis B	Malviya et al. (2014)
28.	Arjunolic acid		Protects against arsenic-induced oxidative injury	Sun et al. (2021)
Tannins				
29.	Punicalagin		Antiinflammatory, antimicrobial, cardioprotective, anticancer, and antioxidant	Čolić et al. (2022)
30.	Punicallin		Antimicrobial and antioxidant	Malviya et al. (2014)
31.	Ellagic acid		Antioxidant, antihepatotoxic, neuroprotective effects, antisteatosic, anti-cholestatic, antihepatocarcinogenic, antifibrogenic, and antiviral properties	Morzelle et al. (2016)
32.	Pedunculagin		Antioxidant and ntifungal	Ruan et al. (2022)
33.	Punicatannin C		Anticancer and antimicrobial	Nazeam et al. (2020)
Flavonoids				
34.	Kaempferol		Antimicrobial, antioxidant, neuroprotective, cardiovascular, and anti-inflammatory	Imran et al. (2019)
35.	Catechin		Anti-inflammatory, neuroprotective, anti-diabetic, antimicrobial, memory enhancer, and hepatoprotective	Baranwal et al. (2022)
36.	Quercetin-3-O-glucoside		Antioxidant, anticancer activity, anti-inflammatory	Nile et al. (2021)
37.	Phloretin		Anticancer and antimicrobial	Nazeam et al. (2020)
38.	Quercitin glycoside		Antimicrobial and anticancer	Nazeam et al. (2020)
Organic acids				
39.	Fumaric acid pent side		Antioxidant	Yazdi and Mrowietz (2008)
40.	Quinic acid		Antiviral, antiaging, anti-nociceptive, analgesic, antioxidant, and anti-diabetic	Morzelle et al. (2016)

## *In silico* analysis of the bioactive compounds found in pomegranate peel for potential applications in drug delivery

*In silico* analysis, including molecular docking studies of pomegranate peel bioactive compounds, can enhance the understanding of their therapeutic potential and their role in drug discovery and delivery. Numerous studies have highlighted the efficacy of these components as drug molecules against various target proteins (Table 3). An investigation assessed the binding efficiency of several bioactive compounds against the SARS-CoV-2 virus. The polyphenolic compounds exhibited stronger and more efficient binding to the M^pro^ protease inhibitors of SARS-CoV-2 when compared to traditional COVID-19 medications such as lopinavir, nelfinavir, and curcumin. Notably, polyphenols such as punicalin, cyanidin-3-glucoside, and quercetin-3-O-rhamnoside formed stable complexes with the inhibitor, positioning pomegranate peel as a promising herbal formulation for COVID-19 prevention (Rakshit et al. 2022).

**Table 3:** Bioactive components of pomegranate peels and their potential as drug molecules against different target proteins

S. No.	Bioactive compound	Target protein	Activity	References
1.	Punicalagin	Tyrosinase	Inhibition of tyrosinase by decrease in the melanin content in B16F10 cells	Yu et al.( 2022)
2.	Punicalagin	Alpha glucosidase	Inhibit α-glucosidase that corresponds to the hypoglycemic effect.	Lu et al.(2024)
3.	Punicalagin	AMPK (Adenosine monophosphate-activated protein kinase)	Colitis: *in vivo* study in DSS-induced colitis.	Liu et al.( 2024)
4.	a) Ellagic acid b) Punicalagin c) Punicalin	a) PTP1β (protein tyrosine phosphatase 1β) b) GFAT (Glutamine-fructose-6-phosphate amidotransferase) c) α-amylase d) α-glucosidase e) RBP-4 (retinol binding protein − 4)	Promising candidates for antidiabetic activity, punicalin was most potent	Gull et al. (2023)
5.	Punicalagin	SrtA (enzyme for virulence in *S.aureus*)	Combating MRSA infections	Song et al.( 2022)
6.	Punicalagin	a) IL-6 (Interleukin-6) b) NF-kB (Nuclear factor-kB) c) TNF-α (tumor necrosis factor-α)	A nti-inflammatory and wound healing	El Nady et al. (2024)
7.	Punicalagin	a) AKT1 (Serine/threonine protein kinase 1) b) ALB (serum albumin) c) IGF1 (insulin like growth factor-1) d) SRC (tyrosinase kinase c-SRC) e) CASP3 (caspase-3) f) EGFR (epidermal growth factor receptor)	Punicalagin interacts with these receptors to mediate peptidoglycan and lipid, reducing the buildup of neurotoxic proteins in the brain and helping to combat Alzheimer’s.	Xu et al.( 2022)
8.	Punicalagin	HSV-2 (Herpes simplex virus)	Anti-viral activity against HSV2.	Arunkumar and Rajarajan (2018)
9.	a) Gallic acid b) Punicalin c) α-Punicalagin d) β-Punicalagin e) Ellagic acid	SARS-CoV-2 S-glycoprotein	Attenuate SARS-CoV-2 S glycoprotein binding ability to ACE2 and shows *in vitro* inhibition activity.	Suručić et al. (2021)
10.	Pomegranate peel	a) Procyanidin b) Delphindin c) Cyanidin	6-phosphogluconate dehydrogenase (6PGD) inhibitory activity *In vitro* breast cancer activity on MCF-7 cell lines and *in vivo* against mice models.	Riaz et al.( 2024)

## Therapeutic properties of pomegranate peel

Pomegranate peels are shown to exhibit therapeutic properties due to their rich content of bioactive components. The various therapeutic activities exhibited include antioxidant, anti-inflammatory, cardiovascular protection, antimicrobial, hepatoprotective, anticancer, and. anti-obesity (Cheng et al. 2023; Siddiqui et al. 2024).

### Anti-oxidant activities

**Fig. 4:**
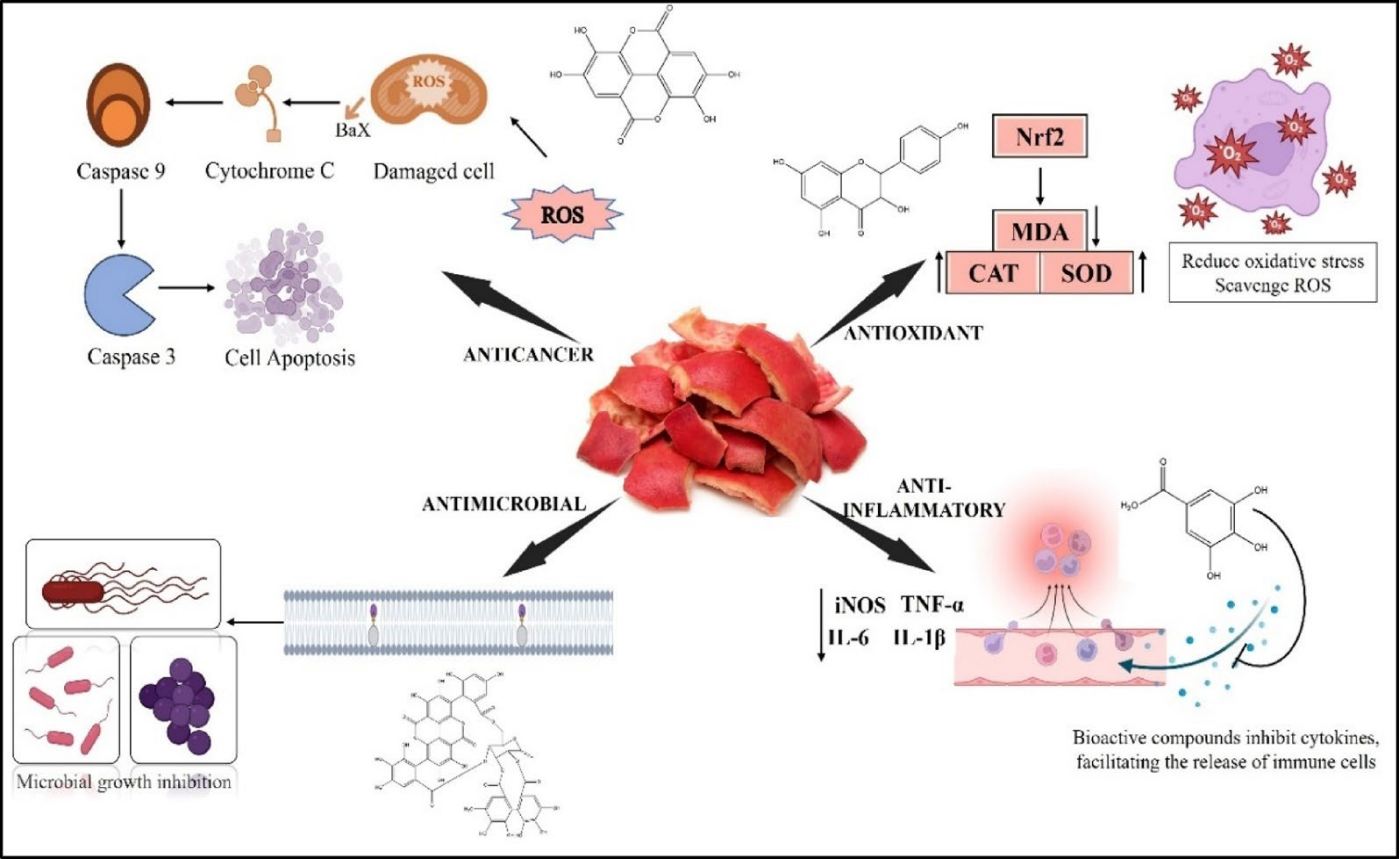
Mechanism of action of pomegranate peels bioactive compounds for therapeutic activities, including anti-inflammatory (inhibition of pro-inflammatory cytokines), antioxidant (Nrf-2 activation), antimicrobial (disrupting the bacterial cell membrane and inhibiting the growth), and anticancer (ROS-mediated apoptosis)

Pomegranate peels are rich in antioxidants, a finding supported by numerous studies (Table 4). In a study by Wu et al. (2019), researchers examined the effects of pomegranate peel polyphenols *in vivo* on mice models that had been immunosuppressed with a cyclophosphamide injection (Wu et al. 2019). The results showed that these polyphenols antioxidant effectiveness varied with its dosage. It reduced malondialdehyde levels in immunosuppressed mice and increased the activity of enzymes such as superoxide dismutase (SOD), glutathione peroxidase, and catalase (CAT), indicating a key mechanism behind its antioxidant effects Fig. 4. Additionally, pomegranate peel extracts were found to have a protective antioxidant effect against hydrogen peroxide-induced reactive oxygen species production at non-cytotoxic concentrations of 1.0 µg/mL and 10 µg/mL, as confirmed by the reduction in levels of malondialdehyde (Mastrogiovanni et al. 2020). Extracts from pomegranate peel may lower levels of oxidized LDL, lipid peroxidation, thiobarbituric acid-reactive substances, and other oxidative markers associated with cardiovascular risk in healthy individuals (Rosenblat et al. 2006; Jing et al. 2019). This highlights the importance of pomegranate peel as a key dietary source of antioxidants.

**Table 4:** Therapeutic potential of pomegranate peels along with the underlying molecular mechanism supported by experimental studies

S.No.	Therapeutic activity	Mechanism of action	Experimental evidence	References
1.	Antioxidant and Anti-inflammatory Activity	Increase in the levels of SOD, CAT, and Glutathione (GSH), while there is a decrease in the levels of Malondialdehyde (MDA) for antioxidant activity. Reduction in expression levels of TNF-α, COX-2, and IL-10 through the NF-κB pathway for anti-inflammatory activity	*In vivo* in Wistar rats	Sayed et al.( 2022)
2.	Antioxidant	Increase in CAT, glutathione peroxidase, and MDA. Reduction in lipid peroxidation.	*In vivo* in rat models	Rak-Pasikowska et al. (2024)
3.	Antioxidant and Liver Protection	Peels exhibit antioxidant activity by reducing the levels of aspartate and alanine transaminases, liver myeloperoxidase, lactate dehydrogenase, and MDA. Lowers lipid globules and hepatic fibrosis.	Randomized human clinical trial	Barghchi et al. (2023)
4.	Modulation of Gut microbiota and Anti-inflammatory activity	Punicalagin-rich peel extract, when administered, shows an increase in bacteria that influence good health. Production of short-chain fatty acids reducing inflammation, urolithin A, and other secondary metabolites	Randomized human clinical trial	Sivamani et al. (2024)
5.	Anti-inflammatory	Suppression of IL-6, TNF-α, iNOS, and COX-2 expression through NF-κB, MAPK signalling pathways.	*In vitro* in RAW 264.7 cells	Li et al. (2022)
6.	Anti-inflammatory	Reduction in levels of C-reactive protein, serum amyloid-A, and total cholesterol	*In vivo* in Albino rats	Salama et al. (2021)

### Anti-inflammatory activities

The pomegranate peel extracts (PPE) exhibit anti-inflammatory activities (Table 4). The activity of peel extracts was studied on RAW264.7 cell lines. The PPE-treated cells produced fewer cytokines by blocking the release of IL-6. This was a dose-dependent impact, with cytokine levels gradually declining as PPE concentrations increased (Feng et al. 2022). Another study found that the significant bioactive substances responsible for the anti-inflammatory qualities were ellagic acid (EA) and punicalagin (PC). Pomegranate peel polyphenols and other components LPS-induced IB phosphorylation, ubiquitination, and degradation, which in turn prevented NF-B activation and p65 nuclear translocation (Du et al. 2019). A study conducted by Mastrogiovanni et al. (2020) emphasizes the anti-inflammatory properties of pomegranate peel water extract. This extract decreases CXCL8 secretion in TNF-stimulated Caco-2 cells and inhibits the expression of pro-inflammatory cytokine genes in LPS-challenged colonic tissues. These findings suggest PPE could be a valuable functional food ingredient for maintaining gastrointestinal tract homeostasis (Mastrogiovanni et al. 2020). This potential effect may be due to the significant reduction in levels of cytokines, such as TNF-α, Interleukins, and Cyclooxygenases, mediated through NF-κB signaling pathways (Fig. 4) (Li et al. 2022).

### Anticancer activities

Cancer significantly contributes to elevated mortality rates in both developed and developing countries (Aneja et al. 2025). Bioactive compounds such as ellagic acid and punicalagin found in pomegranate peel have demonstrated anticancer properties (Mo et al. 2022). Several studies have demonstrated the effect of pomegranate peels on various types of cancer, along with their underlying mechanisms of action (Table 5).

Ellagic acid, a major active component, exhibits antimutagenic, antifibrotic, and antiaging effects. Extensive research on human hepatoma HepG2 cells demonstrates that pomegranate peel polyphenols effectively arrest the cell cycle at the S-phase, modulate levels of reactive oxygen species (ROS), and induce apoptosis. These findings suggest that these polyphenols hold significant potential as a therapeutic agent in the treatment of liver cancer (Teniente et al. 2023). Methanolic extracts of pomegranate peel are known to exhibit therapeutic effects against cancer, demonstrating concentration- and time-dependent reductions in cell proliferation, increased apoptotic cell counts, and modifications in Bax/Bcl-2 expression. Furthermore, the polyphenols present in the peel show antineoplastic activity against cervical cancer by regulating apoptosis, cellular redox balance, and various signalling pathways (Teniente et al. 2023). The peels exhibit therapeutic benefits by increasing the Bax/Bcl-2 ratio, activating caspase 3 and 8, and expression of p53, ultimately leading to apoptosis (Fig. 4).

### Antimicrobial activities

Pomegranate peel has gained interest due to its impressive antimicrobial properties, establishing it as a significant natural resource with various applications. Pomegranate peels demonstrate a broad antimicrobial spectrum activity against numerous bacteria, fungi and moulds.

**Table 5:** Therapeutic potential of pomegranate Peel in cancer, along with the underlying mechanism of action supported by experimental evidence

S.No	Type of cancer	Experimental evidence	Therapeutic action	References
1.	Prostate cancer	Prostate cancer cell lines from humans and mice include the DU145, PC3 and TRAMP-C1, respectively.	Increased Bax/Bcl2 expression ratio. Involved in the mitochondria-mediated intrinsic apoptotic pathway.	Deng et al.( 2017)
2.	Tongue cancer	Cell lines of human tongue squamous cells (HNO-97 and CVCL_D227).	Induce apoptosis and have effects on cancer cell metastasis.	Ahmed et al. (2023)
3.	Cervical cancer	Cervical cancer cell lines (HeLa and SiHa).	Promotes autophagy by activation of ROS-JNK-Bcl2 pathway facilitated by the release of BECN1 from Bcl2.	Xie et al. (2022)
4.	Lung cancer	Hep3B cell line	The *in silico* studies showed that ellagic acid was the most potent compound exhibiting the best docking against the EGFR-TK inhibitor.	Danjolli-Hashani et al. (2025)
5.	Breast cancer	Human MCF-7 cell lines	The peel extract showed apoptosis via caspase-3-independent, caspase-8, and p53-dependent pathways.	Bandara et al. (2024)
6.	Colon cancer	*In vivo* male albino mice	Activity observed due to the reduction of serum levels of Bcl2 and HIF1-α and high antioxidant activity.	Ammar et al. (2023)
7.	Thyroid cancer	*In vivo* in mice models and *in vitro* on BCPAP cell line	Induces apoptosis via mitochondria-mediated intrinsic pathway. The peel extract also downregulated the MMP-9 expression in the cell lines.	Li et al. (2016)

The peels exhibited more antimicrobial activity than the other parts of the plant (Chen et al. 2020). Pomegranate peel extracts show antimicrobial properties, primarily attributed to tannins and flavonoids (Chen et al. 2020). The key bioactive component identified contributing to the antimicrobial activity is reported to be punicalagin (Gosset-Erard et al. 2021).

The antimicrobial action is primarily due to the ability of these bioactive components to disrupt the microbial cell membrane, inhibit crucial enzymes, and prevent microbial proliferation (Bialonska et al. 2009; Sharma and Bisht 2017) (Fig. 4). In a study by Lee et al. (2017), it was demonstrated that pomegranate extracts possess potential antibacterial activity against skin pathogens, including *Staphylococcus aureus* and *Propionibacterium acnes*, with an MIC of 62.5 µg/mL. It was identified that punicalagin and punicalin were the primary bioactive compounds responsible for this significant activity (Lee et al. 2017). A recent study found that pomegranate peel extracts in water show significant antimicrobial effects against *Listeria monocytogenes*. In contrast, butanoic and methanolic extracts inhibit the growth of *Bacillus subtilis*, *Bacillus megaterium*, and *Bacillus sphaericus*. Additionally, it was reported that boiling these extracts caused a decrease in the MIC values (Abu-Niaaj et al. 2024).

### Wound healing activities

Pomegranate peel extracts have demonstrated significant wound-healing potential, attributed to their bioactive components and associated antioxidant activity. One study investigates the underlying mechanisms of this potential by testing PPE on human dermal fibroblast cells *in* *vitro*. The results indicated that PPE upregulated the expression of the Fibronectin gene (FN1). The FN1 gene plays a key role in regulating cellular adhesion and the organization of the extracellular matrix. Furthermore, PPE enhances collagen synthesis and promotes the accumulation of glycosaminoglycans, both of which contribute to strengthening the dermal matrix and facilitating tissue formation (Hashemi Poor et al. 2022). In a recent study, a carbomer-based gel was prepared using PPE for topical applications and wound healing properties. The gels were checked for their antimicrobial activity against *Staphylococcus aureus* and *Candida albicans*. The gel was found to be effective against all the tested strains, and the one with 2.5% w/w of PPE showed maximum potential for wound healing and antimicrobial purposes (Ferreira et al. 2024).

## Pomegranate peel-mediated nanoparticle synthesis

The waste-to-wealth strategy involves using plant waste materials to synthesize nanoparticles, which can be applied across various industries. Nanoparticles have been produced using a variety of metals, metal oxides, organic compounds, and different parts of pomegranate for diverse purposes (Table 6).

**Table 6:** Different types of nanoparticles prepared from parts of pomegranate with their varied applications

Types of nanoparticles	Part of plant used	Applications	References
Iron oxide	Seeds	Textile dye degradation	Bibi et al.( 2019)
Zinc oxide	Peels	Cytotoxicity	Mohamad Sukri et al. (2019)
Gold	Peels	Diabetes nephropathy	Manna et al. (2019)
Titanium oxide	Peels	Water disinfection	Abu-Dalo et al.(2019)
Chitosan	Peel	Food packaging	Cui et al. (2020)
Silver	Leaf	Antimicrobial activity	Swilam and Nematallah ( 2020)
Zinc oxide	Leaves and flowers	Antibacterial activity	Ifeanyichukwu et al. (2020)
Copper oxide	Peels	Cytotoxic effects	Mahmoud et al. (2020)
Gold	Peels	Yogurt formulation and cytotoxicity analysis	Esther Lydia et al. (2020)
Zinc oxide	Peels	Sunscreen formulation	Kokabi and Ebrahimi (2021)
Zinc oxide	Juice	Antioxidant, antimicrobial, and anticorrosive	Ibadi et al. (2022)
Silver-zinc bimetallic	Peel	Antimicrobial and anticancer	(Hashem and El-Sayyad (2023)

**Fig. 5:**
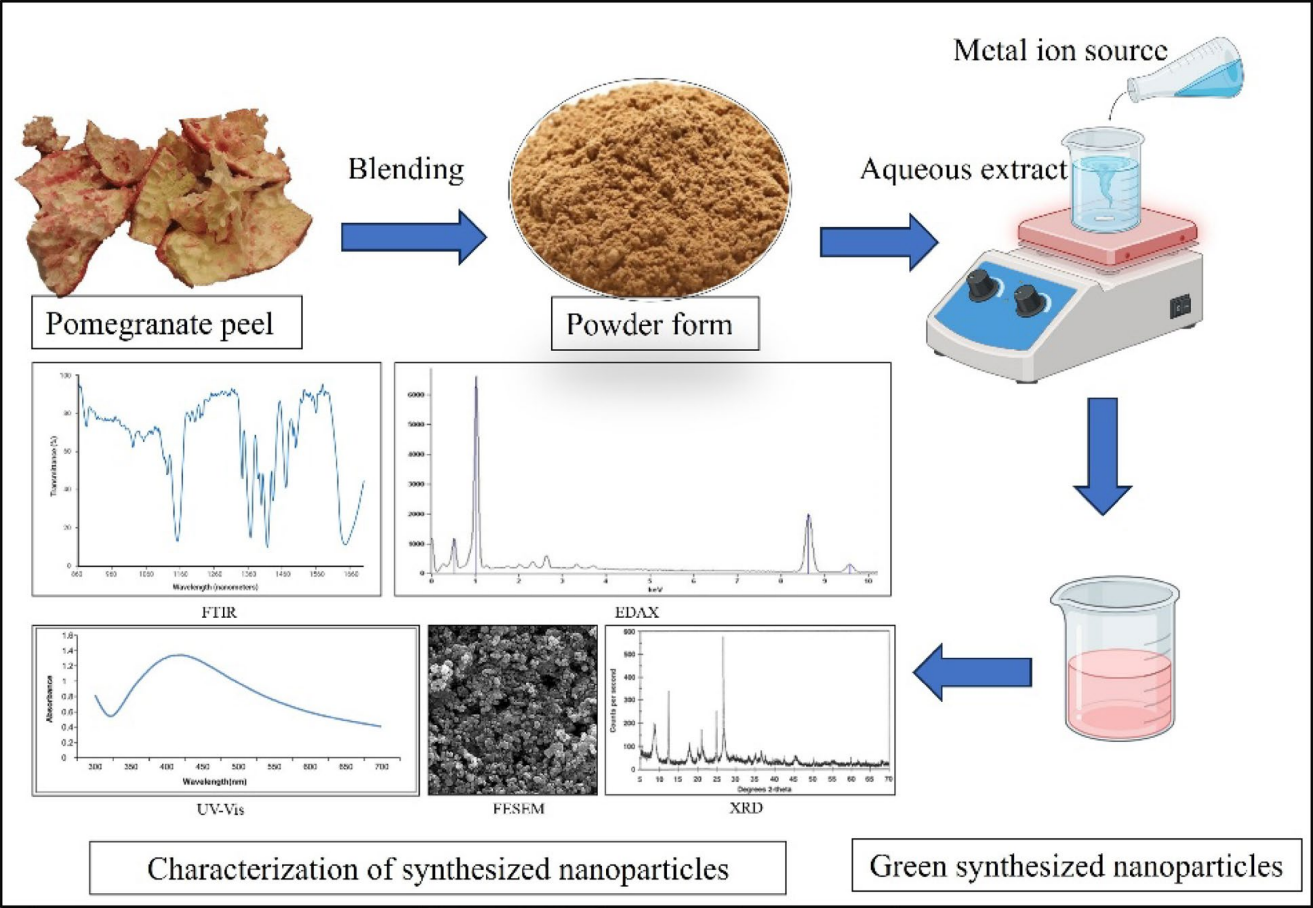
Overview of the green synthesis of nanoparticles mediated by pomegranate peel

In contrast to conventional chemical synthesis, the green synthesis of nanoparticles utilizing plant extracts is more efficient. This process involves combining plant extracts with a source of metal ions, which ultimately determines the type of metal oxide nanoparticles formed. A change in color signifies the successful formation of these nanoparticles, which are subsequently characterized through various analytical techniques. Figure 5 illustrates a general method for synthesizing nanoparticles using pomegranate peel. The bioactive components in pomegranate peels act as capping agents during nanoparticle formation.

This green synthesis method, utilizing pomegranate peel extracts, is notably effective as it avoids harmful chemicals and protects the surrounding environment, thereby offering a cost-effective solution. The affordability, availability of natural plant sources, and use of non-toxic substances contribute to the high tolerability, reproducibility, effectiveness, and biocompatibility of the resulting nanoparticles (Singh et al. 2023a). Pomegranate peel-mediated nanoparticles present a range of biomedical and cosmeceutical applications. In terms of biomedical applications, these nanoparticles exhibit antimicrobial, anticancer, antioxidant, wound-healing, and anti-inflammatory properties, and can also serve as drug carriers and biosensors (Monika et al. 2022). Additionally, their cosmeceutical applications include formulation into creams, soaps, and sunscreens. Compared to conventionally synthesized nanoparticles, those produced through green synthesis provide comparable or enhanced therapeutic benefits, as they utilize the bioactive components within the pomegranate peel as reducing agents (Abdelmigid et al. 2022). Moreover, studies suggest that these green nanoparticles exhibit lower systemic toxicities when properly characterized. However, additional validation through *in vivo* and clinical studies remains essential to confirm their safety (Tavan et al. 2023).

### Gold nanoparticles (AuNPs)

AuNPs are versatile materials with many applications. Gold nanoparticles have numerous applications attributed to their unique mechanical, electrical, thermal, optical, and chemical properties. Additionally, the FDA has approved the use of AuNPs in biomedical applications (Sani et al. 2021).

Manna et al. (2019) used aqueous extracts of pomegranate peel and added them dropwise to the chloroauric acid (HAuCl_4_) solution to synthesize pomegranate peel extract-mediated AuNPs (Manna et al. 2019). Patel et al. (2019) prepared the pomegranate peel extract and combined it with chloroauric acid (1 mM) to synthesize AuNPs. The shift in color from gold to pink signified the formation of gold nanoparticles mediated by pomegranate peel (Patel et al. 2019).

### Silver nanoparticles (AgNPs)

AgNPs are of significant interest due to their prospective applications in catalysis, plasmonics, biological sensors, antimicrobial properties, DNA sequencing, climate change mitigation, water treatment technology, and a variety of other medicinal applications (Rafique et al. 2017). Swilam et al. (2020) mixed pomegranate leaf extracts with a silver nitrate solution in a 5:1 ratio to synthesize silver nanoparticles, and then optimized their synthesis (Swilam and Nematallah 2020). The appearance of a dark brown color confirms the synthesis of AgNPs (Khan et al. 2021). The pomegranate peel-mediated AgNPs are known to have potential antimicrobial activities, with MICs of 25 µg/mL against *Bacillus subtilis*, 50 µg/mL against *S. typhi*, 125 µg/mL against *K. pneumoniae*, and 250 µg/mL against both *E. coli* and *E. faecalis* (Farouk et al. 2023). Additionally, AgNPs have been found to exhibit significant wound-healing properties, thereby demonstrating an anti-inflammatory impact as well (Scappaticci et al. 2021). Since they exhibit explicit antibacterial and anti-inflammatory properties, they can be incorporated into ointments, skin creams, and moisturiser formulations. They can also be used in formulations of creams used for acne treatment and can be incorporated into sunscreens (Ong and Nyam 2022).

### Zinc oxide nanoparticles (ZnONPs)

The plant-mediated synthesis of zinc oxide nanoparticles exhibits several beneficial properties, including antimicrobial, antioxidant, and photocatalytic efficacy. They can also be used in solar cells or antimicrobial coatings as well (Bandeira et al. 2020). For the synthesis of pomegranate peel-mediated ZnO nanoparticles (ZnONPs), any metal salt of zinc can be used. The peel extract was mixed in a 1:20 ratio, and the pH was adjusted to 12. The mixture was then continuously stirred for 2 h at 100 °C. White-coloured powder confirmed the formation of ZnONPs (Shaban et al. 2022). Zinc oxide is often used as a UV filter and is proven to be a potential agent for blocking UVA-type radiation.

### Iron oxide nanoparticles

Iron oxide nanoparticles find extensive applications across diverse fields, including environmental regulation, stem cell tracking, the biomedical sector, tissue engineering, cosmetics, and biosensing. Super magnetism, reduced susceptibility to oxidation, and flexible surface chemistry are some of the physical characteristics of these nanoparticles that make them valuable and versatile (Priya et al. 2021). For synthesizing iron oxide nanoparticles, a solution of FeCl_2_ or FeCl_3_ was used, mixed with an aqueous extract of pomegranate peels. The pH was adjusted by adding NaOH dropwise. The mixture was stirred for 2 h at 1000 rpm and 70 °C. The change in color from orange to black indicates the formation of iron oxide nanoparticles (Ahmed et al. 2023a). Due to their magnetic properties, they are widely used in the technique of Magnetic Resonance Imaging (MRI) (Ansari et al. 2017).

## Pomegranate peel-mediated nanoparticles as an ingredient in skincare formulations

**Fig. 6:**
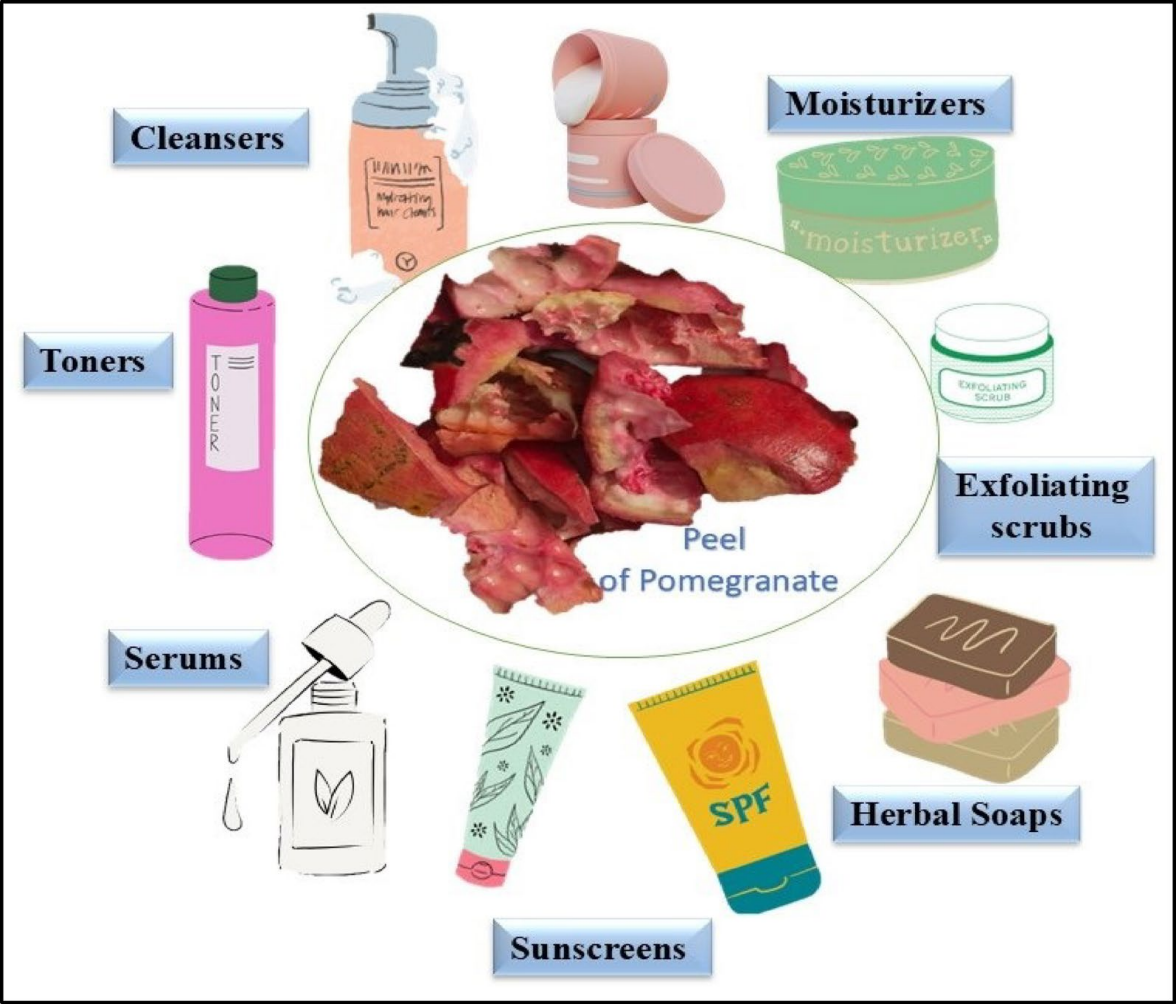
Skincare formulations developed using pomegranate peel-based nanoformulations

Skincare formulations can be explicitly defined as cosmetics for the epidermis of the skin to maintain healthy skin. Skincare products are available in various physical states, including solids, semisolids, and liquids. The primary objectives of using these formulations on the skin include cleansing action (effective removal of microorganisms and dirt from skin), moisturization (establishment of an effective barrier to prevent water losses through the epidermal layer); hydration (maintenance and restoration of the proper fluid balance), and protection of the skin from any external damage from pollution, UV light, dirt, etc. (Salvioni et al. 2021). The incorporation of nanoformulations into skincare formulations enables the controlled release of bioactive components. Various nano skincare formulations include moisturizers, sunscreens, anti-aging creams, cleansers, and anti-acne formulations. These formulations also show a variety of skin benefits (Fig. 6).

Pomegranate peels contain a variety of bioactive compounds that provide numerous benefits for skin health. The presence of ellagic acid was found to reduce skin wrinkles and UV radiation-induced hyperpigmentation (Singh et al. 2018). In an *in vitro* study, it was found that punicalagin influences melanocytes to reduce melanin production. The underlying mechanism may be that humans do not directly absorb punicalagin; instead, it is converted into ellagic acid. This compound has been recognized as a potential inhibitor of the tyrosinase enzyme, which plays a crucial role in the melanin biosynthesis pathway (Rana et al. 2013).

### Creams

Due to their rich bioactive compounds, pomegranate peels have attracted significant attention from the cosmetic industry for use in face creams. The peels are specially recognized for their elevated concentrations of polyphenols, tannins, and flavonoids, which provide antioxidant and anti-inflammatory advantages (Jalal et al. 2018). These compounds play a crucial role in protecting the skin from oxidative stress, reducing inflammation, and promoting overall skin health. Moreover, ellagitannins found in pomegranate peels are associated with potential anti-aging benefits, as they aid in collagen production and enhance skin elasticity (Eghbali et al. 2021). Therefore, incorporating pomegranate peel extracts in various creams offers a natural and botanical approach to skincare, catering to the growing demand for formulations that prioritize both effectiveness and sustainability. Kalouta et al. (2020) prepared a cream composed of natural wax and an emulsifier derived from olive oil. It contained 0.5% pomegranate-encapsulated nanofibers and 16.5% natural and synthetic oils stabilized by an acrylic polymer. Next, the rheological behavior of these creams was investigated to analyze their interaction with the skin (Kalouta et al. 2020). Colloidal ZnO synthesized from *A. vasica* leaves exhibited potential antimicrobial and antioxidant activities, which led to its incorporation in a cold cream formulation that showed antibacterial and antifungal activities against various pathogens causing skin infections and alleviated oxidative stress and cellular damage (Sonia et al. 2017).

### Nanoemulsions for moisturizers

Pomegranate peel offers a rich supply of antioxidants, with a high concentration of gallic acid that helps prevent the formation of free radicals and shields against damaging UV radiation. Nano emulsions are used in various skincare applications, including moisturizers, as they aid in the active delivery of bioactive components with the benefits of stability, solubility, and maintaining their potential benefits (Yadwade et al. 2021). In a study, researchers prepared pomegranate-based seed oil nanoemulsions using the ultrasonic emulsification solvent evaporation method. Ethyl acetate fraction, soy lecithin, and pomegranate seed oil were dissolved in 10 mL of ethyl acetate. The solution was then poured slowly into 40 mL of polysorbate 80. The *in vitro* skin penetration studies were then performed, which indicated that these nanoemulsions helped the polyphenols present in the peel penetrate the skin more effectively and protect it from oxidative damage (Baccarin and Lemos-Senna 2017). The preparation of plant-based nanoemulsions is advantageous as it aids in various skin repair mechanisms. Nanoemulsions are beneficial as they support processes such as collagen synthesis, melanin production, and anti-aging. Incorporating nanoemulsions into moisturizers improves skin hydration by creating a thin film that prevents dryness and by carrying hygroscopic compounds that aid in moisture retention (Romes et al. 2021). Nanoemulsions are said to minimize or eliminate skin irritation as they penetrate directly into the skin through pores and hair follicles without harming healthy tissue. This results in improved skin hydration, an attractive visual appearance, and enhanced stabilization of bioactive ingredients in the product (Naseema et al. 2021).

### Sunscreens

**Fig. 7:**
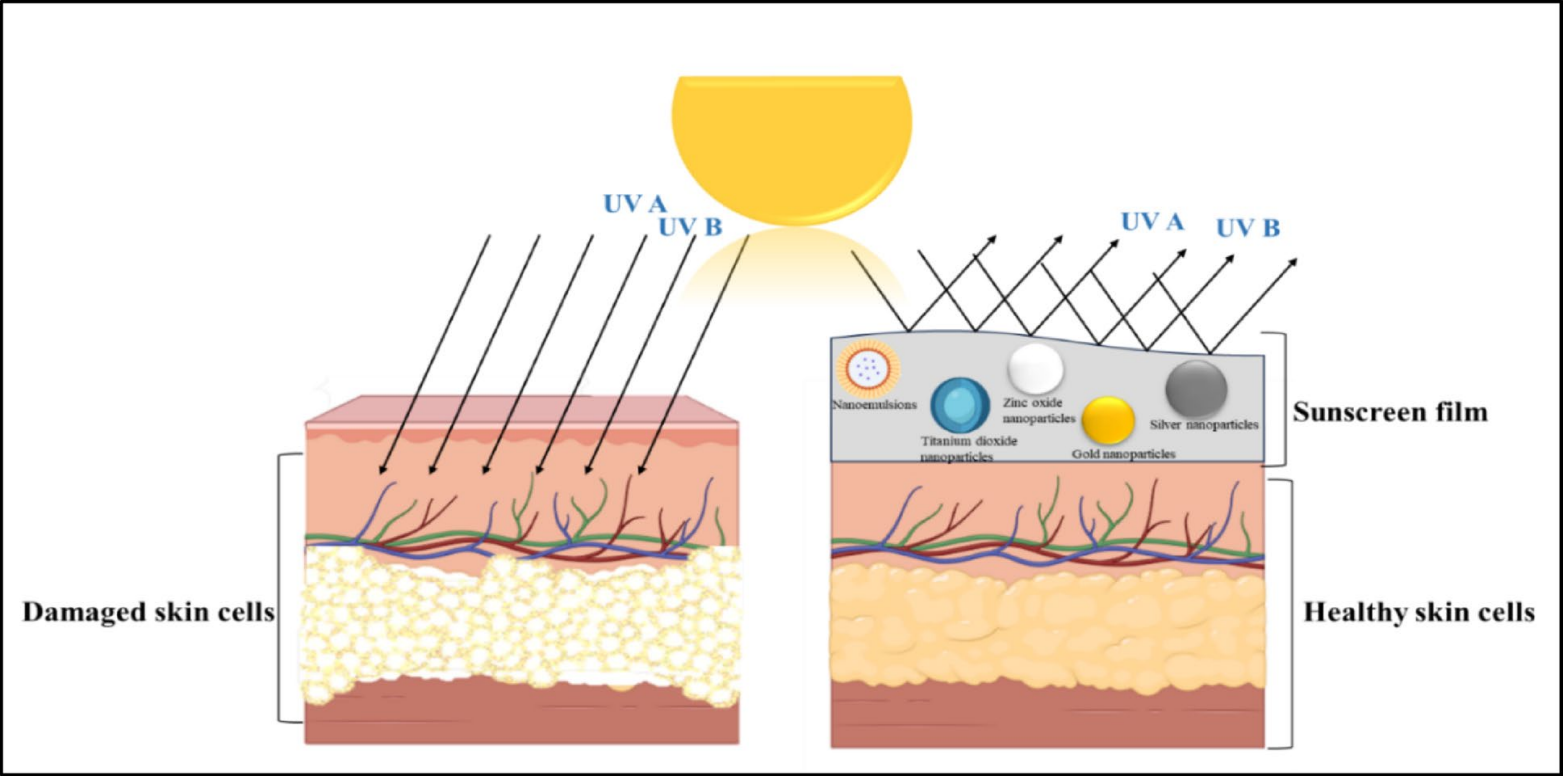
Protective mechanism of sunscreens; the left part indicates the part of the skin that does not have sunscreen application, whereas the nanoparticle-containing sunscreen is applied on the right part. It protects the cells from harmful UV radiation, allowing them to remain healthy

Photoaging refers to the process of gradual skin deterioration resulting from prolonged exposure to the sun. This process involves several changes, such as an increase in pigment heterogeneity, collagen degradation, sunburns, a change in epidermal thickness, and mutagenesis of melanocytes and keratinocytes in the skin (Guan et al. 2021). As per the report, 80% of skin ageing is caused by skin exposure to UV light. The application of a photoprotective agent, such as sunscreen, can be the most effective measure of prevention (Flament et al. 2013). Opting for broad-spectrum sunscreens is ideal, as they protect against both harmful UVA and UVB rays. Sunscreens are primarily categorized into two types: physical and chemical. Physical sunscreens block and scatter UV radiation from the skin (Fig. 7).

The active ingredients are titanium dioxide (TiO_2_), zinc oxide (ZnO_2_), and minerals; these ingredients are often incorporated in their nano forms in the cream, which offers several advantages. On the other hand, chemical sunscreens work by absorbing sunlight, and their formulations are usually alcohol-based or lipophilic, containing active ingredients such as octisalate, oxybenzone, octocrylene, octinoxate, homosalate, and avobenzone (Yamada et al. 2020). Therefore, selecting the correct type of sunscreen is crucial for protecting the skin from harmful UV radiation. Both titanium and zinc oxide nanoparticles are incorporated in the physical sunscreen to achieve maximum benefits of UV protection. These particles are smaller in size, resulting in a more even application on the skin and providing an increased surface area for the reflection of UV radiation. These are often odourless and colourless, and do not form any white residue upon application to the skin (Lyu et al. 2022).

### Skin cleansers

The hydrophobic skin layers contain sebaceous and sweat glands, which accumulate endogenous and exogenous contaminants. For maintaining skin health, it is essential to remove dust, irritants, dead skin, and odour. Proper cleansing using a good cleanser helps eliminate all the resident microbes and allergens that accumulate on the skin surface (Salvioni et al. 2021). Instead of using basic, conventional soaps and cleansers, innovative nanoformulations, such as nanoemulsions and metal-based nanoparticles, can be explored. When prepared using pomegranate peel extracts, these nanoformulations not only enhance cleansing but also provide beneficial effects, including antimicrobial and anti-inflammatory properties. Several studies have been conducted that suggest incorporating silver nanoparticles into formulations such as soaps, hand washes, and gels. These formulations claimed to have antimicrobial properties, fight acne, and sunburned skin, and other benefits as well (Nafisi and Maibach 2017).

## Pharmacokinetic analysis of the bioactive components in pomegranate peels

The biological activity and potential of compounds as drug candidates are profoundly influenced by their molecular characteristics. In this study, we systematically evaluated 20 bioactive compounds derived from pomegranate peels, analysing their Absorption, Distribution, Metabolism, and Excretion (ADME) profiles using the http://www.swissadme.ch/tool. This analysis not only enhances our understanding of the pharmacokinetics of these compounds but also their drug-likeness. Among the selected compounds, notable entities such as gallic acid, caffeic acid, isohydroxymatairesinol, corosolic acid, phloretin, catechin, kaempferol, ellagic acid, arjunolic acid, and asiatic acid exhibited high gastrointestinal absorption, suggesting potential efficacy upon ingestion, which could contribute positively to human health. In terms of distribution, factors such as P-glycoprotein (P-gp) efflux, penetration of the blood-brain barrier (BBB), and implications for the central nervous system (CNS) were also assessed. The analysis indicated that none of the evaluated compounds could cross the BBB, suggesting minimal effects on the central nervous system, an essential consideration for safety in therapeutic applications. Additionally, several compounds, including punicacortein C, granatin A, isohydroxymatairesinol, corosolic acid, catechin, pedunculagin, punicalin, punicalagin, and asiatic acid, were identified as P-gp substrates; their activity may limit gastrointestinal absorption, resulting in restricted distribution throughout the body. For metabolic profiling, the interaction of these compounds with cytochrome P450 enzymes (CYPs) was examined. Among all the selected bioactive compounds, two flavonoids, phloretin and kaempferol exhibited inhibitory effects on CYP enzymes. This lack of inhibition implies a lower risk of drug-drug interactions, thereby positioning these compounds as safer options for incorporation into dietary or therapeutic products. In terms of excretion, the profiling of water solubility and lipophilicity provided insights into the excretion pathways of these compounds. Such characteristics are vital for understanding the behaviour of these bioactive components within the human body and their potential long-term effects (Table S1).

Other significant factors in the ADME profiling, which suggest the feasibility of incorporating these bioactives into skincare formulations, are illustrated in Table 7. These crucial factors include molecular weight, Log Kp value, and TPSA (Topological Polar Surface Area) values. The bioactive compounds isolated from pomegranate peels were categorized into major groups, including phenolic acids, tannins, flavonoids, and triterpenoids, and their characteristics were examined in relation to skincare applications. For a compound to be primed for effective transdermal absorption, it should have a molecular weight of less than 500 Da. The Total Polar Surface Area (TPSA) values act as a valuable compass in gauging a compound’s ability to navigate and penetrate biological barriers, especially in the realm of absorption (Daina et al. 2017). Additionally, the Log Kp value serves as an insightful indicator of the skin permeability coefficient of bioactive molecules; a Log Kp exceeding (− 2.5) clearly reveals that the compound with low skin permeability, presents a challenge for its absorption through the skin’s protective layers (Vijayakumar et al. 2022).

On further investigations, the following observations have been made. The major polyphenolic acids, such as gallic, brevifolincarboxylic, and caffeic acid, have molecular weights less than 500Da (170–292 Da), with moderate LogKp values (-6.58 to -8.38 cm/s). The values of these compounds demonstrate excellent permeability, making them promising antioxidant candidates that can be utilised in sunscreen formulations, lotions, and other daily-use skincare products. The tannin compounds like punicalagin, pedunculagin, punicalin, granatin A, punicacortein C, and causuariin show high molecular weights ranging from 634.5–1084.72 g/mol and LogKp values ranging from − 10.33 to -12.46 cm/s. The permeability of these compounds through the skin is significantly limited due to their relatively high molecular weight. Their low permeability makes them ideal for topical skincare formulations with localized application and antimicrobial properties, such as soap formulations (Fares et al. 2023). A study shows the potential of tannic acidas an anti-photoaging agent (Daré et al. 2020). For flavonoids, catechin, phloretin, and kaempferol, the molecular weight of the compounds is in the range of 270–290 g/mol, and the LogKp value ranges from -6.11 to -7.82 7.82 cm/s. These flavonoid compounds play protective roles in UV protection and can therefore be incorporated into sunscreens. They also exhibit protective activities against collagen and elastin degradation (Čižmárová et al. 2023). The triterpenoid compounds, such as ursolic acid, arjunolic acid, and corosolic acid, have an optimum molecular weight between 450 and 490 g/mol. LogKp value − 3.87 to 5.23 cm/s exhibiting dermal potential. These compounds exhibit anti-aging, antioxidant, anti-inflammatory, scar-reducing, and wound-healing properties (Min et al. 2024). Although there is limited information regarding the toxicity associated with the consumption and topical application of pomegranate peel, a study was conducted to assess its toxicity using BALB mice. The results showed no toxic effects, clinical signs, or histopathological changes in the epithelial cells of the larynx, tongue, and trachea. No adverse effects or mortality were reported in the mouse models. Intradermal injections were performed to evaluate skin allergies, yielding negative test results, which further indicated the lack of toxicity of the peels (Jahromi et al., 2015). Another research suggests that when consumed in moderation, pomegranate peel is generally safe and does not pose toxic effects. However, it is essential to note that high doses of peel extracts can be harmful, making it necessary to evaluate and use appropriate concentrations before consumption (Singh et al. 2023b). Additionally, these peels and their bioactive components have demonstrated no toxicological effects upon topical application, making them entirely safe for use. They possess significant photoprotective potential against UV radiation and can be incorporated into skincare products to promote healthy, disease-free skin (Tumbarski et al. 2025). Future research should focus more on the toxicological effects related to the consumption and topical application of peels.

**Table 7:** ADME profiling of the bioactive compounds identified from pomegranate peel

S.No.	Bioactive compound	Molecular formula	Molecular weight	Log S	TPSA	log Kp (cm/s)	Bioavailability radar
1	Gemin D	C_27_H_22_O_18_	634.45	− 3.53	318.50 Å²	− 10.33 cm/s	
2	Causuariin	C_34_H_24_O_22_	784.54	− 5.00	388.42 Å²	− 11.04 cm/s	
3	Gallic acid	C_7_H_6_O_5_	170.12	− 1.64	97.99 Å²	− 6.84 cm/s	
4	Caffeic acid	C_9_H_8_O_4_	180.16	− 1.89	77.76 Å²	− 6.58 cm/s	
5	Punicacortein C	C_48_H_28_O_30_	1084.72	− 7.43	529.76 Å²	− 12.29 cm/s	
6	Granatin A	C_34_H_24_O_22_	784.54	− 3.73	374.26 Å²	− 12.46 cm/s	
7	Isohydroxymatairesinol	C_20_H_22_O_7_	374.38	− 3.45	105.45 Å²	− 7.06 cm/s	
8	β− sitosterol	C_29_H_50_O	414.71	− 7.90	20.23 Å²	− 2.20 cm/s	
9	Ursolic acid	C_30_H_48_O_3_	456.70	− 7.23	57.53 Å²	− 3.87 cm/s	
10	Corosolic acid	C_30_H_48_O_4_	472.70	− 6.72	77.76 Å²	− 4.66 cm/s	
11	Phloretin	C_15_H_14_O_5_	274.27	− 3.37	97.99 Å²	− 6.11 cm/s	
12	Catechin	C_15_H_14_O_6_	290.27	− 2.22	110.38 Å²	− 7.82 cm/s	
13	Kaempferol	C_15_H_10_O_6_	286.24	− 3.31	111.13 Å²	− 6.70 cm/s	
14	Pedunculagin	C_34_H_24_O_22_	784.54	− 5.61	377.42 Å²	− 10.42 cm/s	
15	Ellagic Acid	C_14_H_6_O_8_	302.19	− 2.94	141.34 Å²	− 7.36 cm/s	
16	Punicalin	C_34_H_22_O_22_	782.53	− 4.88	385.24 Å²	− 11.28 cm/s	
17	Punicalagin	C_48_H_28_O_30_	1084.72	− 8.50	518.76 Å²	− 11.67 cm/s	
18	Arjunolic Acid	C_30_H_48_O_5_	488.70	− 6.42	97.99 Å²	− 5.13 cm/s	
19	Brevifolincarboxylic acid	C_13_H_8_O_8_	292.20	− 1.67	145.27 Å²	− 8.38 cm/s	
20	Asiatic Acid	C_30_H_48_O_5_	488.70	− 6.33	97.99 Å²	− 5.23 cm/s	

## Conclusion

The industrial sector stands to gain significantly from the bioactive components present in pomegranate peels, a nutrient-rich byproduct. The presence of the bioactive compounds make the peel waste a valuable element for applications in healthcare and skincare. Key bioactive compounds contributing to the therapeutic properties and photo-protective activity include specific hydrolysable tannins, such as ellagic acid and punicalagin. Moreover, these bioactives found in the peels act as effective reducing and capping agents in the synthesis of nanoparticles. Incorporating these green-synthesised nanoparticles into skincare products represents an innovative strategy that supports a circular economy approach. Pharmacokinetic studies of the bioactive molecules in pomegranate peels reveal their absorption and skin permeability values, which are beneficial for formulating skincare products. These studies also demonstrate the complete safety of these molecules, indicating their potential for inclusion in advanced nanoformulations at specific concentrations. Future prospects can concentrate on optimizing these nanoformulations for enhanced skin absorption. However, the detailed *in vitro*, *in vivo*, and clinical investigations of these formulations are recommended. Contraindications to the toxicological potential of overuse and overdose of peel extracts in formulations are particular challenges that cannot be overlooked.

## Supplementary Information

Below is the link to the electronic supplementary material.

Supplementary Material 1

## Data Availability

The article and/or supplemental materials contain the datasets that support the findings of this work.

## Abbreviations

LDL: Low-density lipid
PPE: Pomegranate peel extract
IL: Interleukin
EA: Ellagic acid
PC: Punicalagin
LPS: Lipopolysaccharide
TNF: Tumor necrosis factor
NF: Necrosis factor
ROS: Reactive oxygen species
Gold nanoparticles: AuNPs
Silver nanoparticles: AgNPs
FDA: Food and drug administration
MIC: Minimum inhibitory concentration
PRISMA: Preferred Reporting Items for Systematic Reviews and Meta-Analyses
ZnONPs: Zinc oxide nanoparticles
UV: Ultraviolet
ADME: Absorption, Distribution, Metabolism, and Excretion
SDGs: Sustainable Development Goals
DPPH: 2,2-diphenyl-1-picrylhydrazyl
AMPK: Adenosine monophosphate activated protein kinase
PTP1β: Protein tyrosine phosphatase 1β
RBP: retinol binding protein
SrtA: Sortase A
IL6: Interleukin-6
GFAT: Glutamine fructose-6-phosphate amidotransferase
NF kB: Nuclear factor-kB
AKT1: Serine/threonine protein kinase 1
ALB: Serum albumin
IGF1: Insulin like growth factor 1
SRC: Tyrosinase kinase c SRC
CASP3: Caspase-3
HSV: Herpes simplex virus
6PGD: 6-Phosphogluconate dehydrogenase
FN1: Fibronectin gene
EGFR: Epidermal growth factor receptor
HPLC: High Performance Liquid Chromatography
NO: Nitric oxide
