# Supplementary material for: Waste valorization utilizing green nanotechnology: a sustainable approach for pomegranate peel agro wastes in skincare formulations
Source: Bioresour Bioprocess. 2025 Dec 24;12(1):152. doi: 10.1186/s40643-025-00958-6 (PMC12738524; doi:10.1186/s40643-025-00958-6)
Supplement: Supplementary file 1 — Supplementary Material 1 [file 40643_2025_958_MOESM1_ESM.docx]

Table S1: ADMET Profiling of the bioactive compounds identified from pomegranate peel

| **ADME Parameters** | | **Gemin D** | | **Causuariin** | | **Gallic acid** | | **Caffeic acid** | **Punicacortein C** | | **Granatin A** | | **Isohydroxymatairesinol** | | **β-sitosterol** | | **Ursolic acid** | **Corosolic acid** | |
| --- | --- | --- | --- | --- | --- | --- | --- | --- | --- | --- | --- | --- | --- | --- | --- | --- | --- | --- | --- |
| **Molecular formula** | | C_27_H_22_O_18_ | | C_34_H_24_O_22_ | | C_7_H_6_O_5_ | | C_9_H_8_O_4_ | C_48_H_28_O_30_ | | C_34_H_24_O_22_ | | C_20_H_22_O_7_ | | C_29_H_50_O | | C_30_H_48_O_3_ | C_30_H_48_O_4_ | |
| **Molecular weight** | | 634.45 | | 784.54 | | 170.12 | | 180.16 | 1084.72 | | 784.54 | | 374.38 | | 414.71 | | 456.70 | 472.70 | |
| **H-bond acceptor** | | 18 | | 22 | | 5 | | 4 | 30 | | 22 | | 7 | | 1 | | 3 | 4 | |
| **H-bond donor** | | 11 | | 14 | | 4 | | 3 | 18 | | 12 | | 3 | | 1 | | 2 | 3 | |
| **Fraction Csp3** | | 0.19 | | 0.18 | | 0.00 | | 0 | 0.12 | | 0.26 | | 0.35 | | 0.93 | | 0.90 | 0.90 | |
| **Molar refractivity** | | 143.08 | | 176.79 | | 39.47 | | 47.16 | 251.09 | | 171.31 | | 97.29 | | 133.23 | | 136.91 | 138.08 | |
| **No. of rotatable bonds** | | 6 | | 1 | | 1 | | 2 | 1 | | 0 | | 6 | | 6 | | 1 | 1 | |
| **Log S** | | -3.53 | | -5.00 | | -1.64 | | -1.89 | -7.43 | | -3.73 | | -3.45 | | -7.90 | | -7.23 | -6.72 | |
| **Consensus Log P_o/w_** | | -1.03 | | -0.80 | | 0.21 | | 0.93 | -0.41 | | -2.71 | | 2.03 | | 7.24 | | 5.93 | 5.06 | |
| **TPSA** | | 318.50 Å² | | 388.42 Å² | | 97.99 Å² | | 77.76 Å² | 529.76 Å² | | 374.26 Å² | | 105.45 Å² | | 20.23 Å² | | 57.53 Å² | 77.76 Å² | |
| **GI absorption** | | Low | | Low | | High | | High | Low | | Low | | High | | Low | | Low | High | |
| **BBB permeant** | | NO | | NO | | NO | | NO | NO | | NO | | NO | | NO | | NO | NO | |
| **Pgp substrate** | | YES | | YES | | NO | | NO | YES | | YES | | YES | | NO | | NO | YES | |
| **CYP1A2 inhibitor** | | NO | | NO | | NO | | NO | NO | | NO | | NO | | NO | | NO | NO | |
| **CYP2C19 inhibitor** | | NO | | NO | | NO | | NO | NO | | NO | | NO | | NO | | NO | NO | |
| **CYP2C9 inhibitor** | | NO | | NO | | NO | | NO | NO | | NO | | NO | | NO | | NO | NO | |
| **CYP2D6 inhibitor** | | NO | | NO | | NO | | NO | NO | | NO | | NO | | NO | | NO | NO | |
| **CYP3A4 inhibitor** | | NO | | NO | | NO | | NO | NO | | NO | | NO | | NO | | NO | NO | |
| **log Kp (cm/s)** | | -10.33cm/s | | -11.04cm/s | | -6.84cm/s | | -6.58cm/s | -12.29cm/s | | -12.46cm/s | | -7.06cm/s | | -2.20cm/s | | -3.87cm/s | -4.66cm/s | |
| **Lipinski** | | NO  3 violations | | NO  3 violations | | YES | | YES | NO  3 violations | | NO  3 violations | | YES | | YES  1 violation | | YES  1 violation | YES  1 violation | |
| **Bioavailability Score** | | 0.17 | | 0.17 | | 0.56 | | 0.56 | 0.17 | | 0.17 | | 0.55 | | 0.55 | | 0.85 | 0.56 | |
| **Leadlikeness** | | NO | | NO | | NO | | NO | NO | | NO | | NO | | NO | | NO | NO | |
| **Synthetic Accessibility** | | 5.70 | | 7.31 | | 1.22 | | 1.81 | 8.68 | | 7.73 | | 3.90 | | 6.30 | | 6.21 | 6.34 | |
| **Bioavaiblity radar** | | 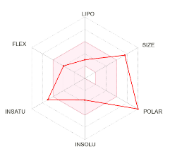 | | 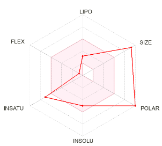 | | 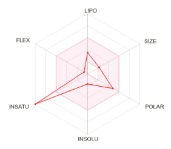 | | 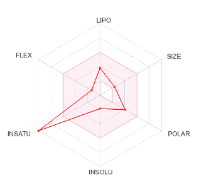 | 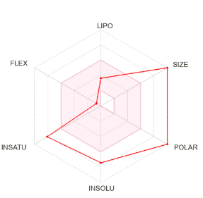 | | 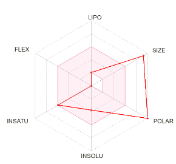 | | 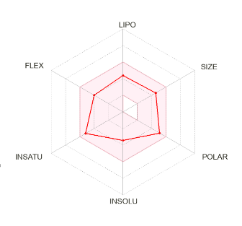 | | 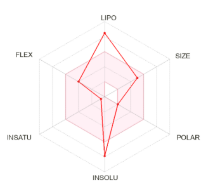 | | 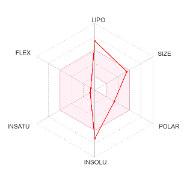 | 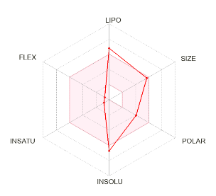 | |
| **ADME Parameters** | **Phloretin** | | **Catechin** | | **Kaempferol** | | **Pedunculagin** | | **Ellagic Acid** | **Punicalin** | | **Punicalagin** | | **Arjunolic Acid** | | **Brevifolincarboxylic acid** | | | **Asiatic Acid** |
| **Molecular formula** | C_15_H_14_O_5_ | | C_15_H_14_O_6_ | | C_15_H_10_O_6_ | | C_34_H_24_O_22_ | | C_14_H_6_O_8_ | C_34_H_22_O_22_ | | C_48_H_28_O_30_ | | C_30_H_48_O_5_ | | C_13_H_8_O_8_ | | | C_30_H_48_O_5_ |
| **Molecular weight** | 274.27 | | 290.27 | | 286.24 | | 784.54 | | 302.19 | 782.53 | | 1084.72 | | 488.70 | | 292.20 | | | 488.70 |
| **H-bond acceptor** | 5 | | 6 | | 6 | | 22 | | 8 | 22 | | 30 | | 5 | | 8 | | | 5 |
| **H-bond donor** | 4 | | 5 | | 4 | | 13 | | 4 | 13 | | 17 | | 4 | | 4 | | | 4 |
| **Fraction Csp3** | 0.13 | | 0.20 | | 0.00 | | 0.18 | | 0.00 | 0.18 | | 0.12 | | 0.90 | | 0.15 | | | 0.90 |
| **Molar refractivity** | 74.02 | | 74.33 | | 76.01 | | 176.55 | | 75.31 | 180.45 | | 250.86 | | 138.98 | | 68.18 | | | 139.24 |
| **No. of rotatable bonds** | 4 | | 1 | | 1 | | 0 | | 0 | 0 | | 0 | | 2 | | 1 | | | 2 |
| **Log S** | -3.38 | | -2.22 | | -3.31 | | -5.61 | | -2.94 | -4.88 | | -8.05 | | -6.42 | | -1.67 | | | -6.33 |
| **Consensus Log P_o/w_** | 1.93 | | 0.83 | | 1.58 | | -0.33 | | 1.00 | -0.83 | | 0.07 | | 4.52 | | 0.04 | | | 4.45 |
| **TPSA** | 97.99 Å² | | 110.38 Å² | | 111.13 Å² | | 377.42 Å² | | 141.34 Å² | 385.24 Å² | | 518.76 Å² | | 97.99 Å² | | 145.27 Å² | | | 97.99 Å² |
| **GI absorption** | High | | High | | High | | Low | | High | Low | | Low | | High | | Low | | | High |
| **BBB permeant** | NO | | NO | | NO | | NO | | NO | NO | | NO | | NO | | NO | | | NO |
| **Pgp substrate** | NO | | YES | | NO | | YES | | NO | YES | | YES | | YES | | NO | | | YES |
| **CYP1A2 inhibitor** | YES | | NO | | YES | | NO | | YES | NO | | NO | | NO | | NO | | | NO |
| **CYP2C19 inhibitor** | NO | | NO | | NO | | NO | | NO | NO | | NO | | NO | | NO | | | NO |
| **CYP2C9 inhibitor** | YES | | NO | | NO | | NO | | NO | NO | | NO | | NO | | NO | | | NO |
| **CYP2D6 inhibitor** | NO | | NO | | YES | | NO | | NO | NO | | NO | | NO | | NO | | | NO |
| **CYP3A4 inhibitor** | YES | | NO | | YES | | NO | | NO | NO | | NO | | NO | | NO | | | NO |
| **log Kp (cm/s)** | -6.11cm/s | | -7.82cm/s | | -6.70cm/s | | -10.42cm/s | | -7.36cm/s | -11.28cm/s | | -11.67cm/s | | -5.13cm/s | | -8.38cm/s | | | -5.23cm/s |
| **Lipinski** | YES | | YES | | YES | | NO  3 violations | | YES | NO  3 violations | | NO  3 violations | | YES | | YES | | | YES |
| **Bioavailability Score** | 0.55 | | 0.55 | | 0.55 | | 0.17 | | 0.55 | 0.17 | | 0.17 | | 0.56 | | 0.56 | | | 0.56 |
| **Leadlikeness** | YES | | YES | | YES | | NO | | YES | NO | | NO | | NO | | YES | | | NO |
| **Synthetic Accessibility** | 1.88 | | 3.50 | | 3.14 | | 6.68 | | 3.17 | 6.74 | | 8.18 | | 6.45 | | 3.54 | | | 6.56 |
| **Bioavaiblity radar** | 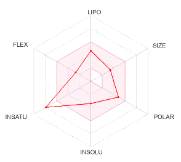 | | 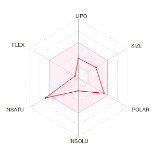 | | 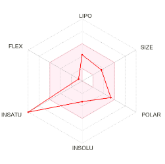 | | 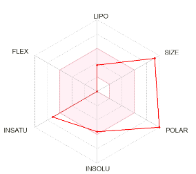 | | 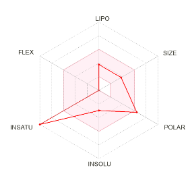 | 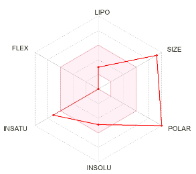 | | 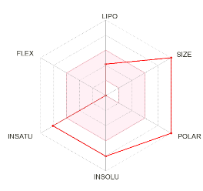 | | 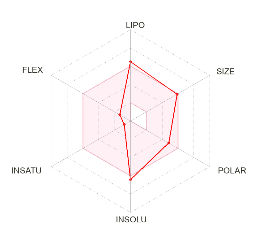 | | 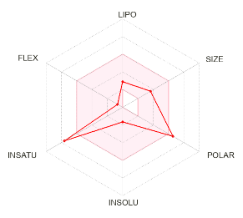 | | | 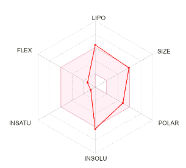 |
